# Natural and Man-Made Cyclic Peptide-Based Antibiotics

**DOI:** 10.3390/antibiotics12010042

**Published:** 2022-12-27

**Authors:** Shian Lai, Quan Zhang, Lin Jin

**Affiliations:** 1 Small Molecule Drugs Sichuan Key Laboratory, Institute of Materia Medica, School of Pharmacy, Chengdu Medical College, Chengdu 610500, China; 2 Department of Molecular Chemistry and Biochemistry, Faculty of Science and Engineering, Doshisha University, Kyotanabe 610-0394, Japan; 3 College of Veterinary Medicine, Shanxi Agricultural University, Jinzhong 030801, China

**Keywords:** antibiotics, antimicrobial peptides, cyclic peptides, peptide design

## Abstract

In recent years, an increasing number of drug-resistant bacterial strains have been identified due to the abuse of antibiotics, which seriously threatens human and animal health. Antimicrobial peptides (AMPs) have become one of the most effective weapons to solve this problem. AMPs have little tendency to induce drug resistance and have outstanding antimicrobial effects. The study of AMPs, especially cyclic peptides, has become a hot topic. Among them, macrocyclic AMPs have received extensive attention. This mini-review discusses the structures and functions of the dominant cyclic natural and synthetic AMPs and provides a little outlook on the future direction of cyclic AMPs.

## 1. Introduction

Antibiotic resistance is considered by the World Health Organization to be one of the three most important major global public health challenges of the 21st century [1]. Bacterial resistance occurs as a result of antibiotic misuse and long-term antibiotic use, including unnecessary antibiotic use and nonprescribed antibiotic use. Agricultural practices include growth promoters and the ease of access to these drugs in developing countries without proper medical prescriptions [1]. Thus, the development of novel antibiotics or new therapeutic regimens is needed to address this problem. Antimicrobial peptides (AMPs) are considered to be an important alternative to multidrug-resistant and pandrug-resistant bacteria.

AMPs, also known as peptide antibiotics, are a class of peptides with broad-spectrum antibacterial activity. It is a class of small short peptides with broad-spectrum antibacterial activity and an important molecular barrier to the host’s natural immune defense system against exogenous pathogens [2]. AMPs usually contain only 9 to 60 amino acids. Wang [3] analyzed 2722 AMPs included in the APD3 database and found that approximately 90% of the AMPs were less than 50 amino acids in length. Among them, the length of functional peptides was concentrated in 21–30 amino acids. Two major classes of AMPs, defensins and cathelicidins, have been identified in most vertebrates. Most of them have remarkable broad-spectrum anti-gram-negative (G-) and gram-positive (G+) activities in vitro [4].

AMPs have great potential for application. First, the advantage of AMPs is that they generally have a sufficient amount of positive charge accompanied by hydrophobicity. AMPs bind to negatively charged biological membranes under electrostatic action and penetrate and disrupt the membrane structure before causing cell death [2]. In addition, unlike the single-target bactericidal principle of traditional antibiotics, AMPs can perform multitarget destruction in pathogens, which can greatly reduce the generation of drug-resistant bacteria [5]. Third, they have broad-spectrum antimicrobial properties. AMPs inhibit bacteria, fungi and viruses and are particularly effective in killing drug-resistant bacteria [6]. Therefore, AMPs are safe and less likely to cause bacterial resistance than conventional antibiotics [6]. It is considered one of the best alternatives to antibiotics in the future. Thousands of AMPs have been identified, and they are classified into four broad categories based on their overall structures: α-helix, β-sheet, extended and cyclic peptides [7]. The next sections focus on the research and development of cyclic peptides.

## 2. Advantages of Cyclic Peptides

**Figure 1:**
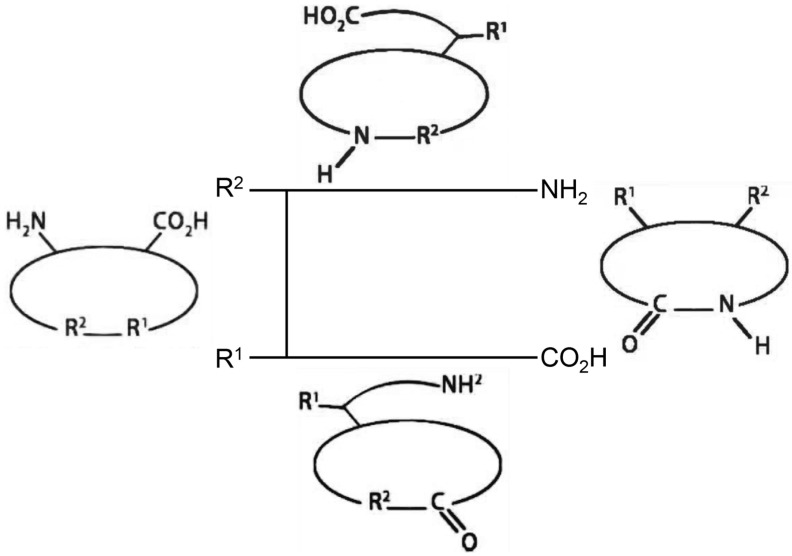
Four types of cyclic peptides in general. Figure was adapted in accordance with Ramadhani et al. [8].

Cyclic peptides are a class of cyclic compounds with specific structures, broad biological activities and unique mechanisms of action. Usually, several amino acids in a specific sequence can form a cyclic peptide. These amino acids are interconnected by amide or other chemically stable bonds between the C– and N–termini of the peptide sequence or between the head and tail (Figure 1) [8]. The ongoing research has brought forward the potential of cyclic peptides. Cyclic peptides contain a cyclic sequence of bonds and have advantages due to their target selectivity [8]. As shown in Figure 1, most of cyclic peptides are resisted to protease enzymes and have better stability against extracellular targets [8].

Cyclic peptides are defined as a special class of natural products. Cyclic peptides can be obtained from natural sources, including plants, algae, sponges, fungi, bacteria and mammals [9]. For example, RA–V, a cyclic hexapeptide, has been isolated and characterized from *Rubiae radix*, showing antitumor activity [10,11]. There are many cyclic peptides, such as cyclosporins, diketopiperazines and sansalvamides, in extracts of the marine fungus *Fusarium oxysporum* [12,13]. Wewakazole B, a cytotoxic cyanobactin, is another cyclic peptide isolated from the Red Sea marine cyanobacterium *Moorea producens* [14]. In addition to the abovementioned naturally derived cyclic peptides, many cyclic peptides, such as bacteriocins, are chemically synthesized [15].

Generally, infections stimulate the production of inflammatory cytokines, and antibiotics may have immunomodulatory properties and are frequently used for their immunomodulatory and antimicrobial properties. Some of the cyclic peptides and their derivates have remarkable immunomodulatory effects that may extend their applications. For example, the novel cyclic lipopeptide antibiotic daptomycin, has good tissue penetration and is more effective in biofilm [16]. It was reported that daptomycin causes immunomodulation-suppressing cytokine production after methicillin-resistant *Staphylococcus aureus* infection [17]. Hidradenitis suppurativa is a chronic inflammatory skin disease characterized by painful nodules, abscesses and fistulas [18]. Researchers showed that the use of dalbavancin is an effective and well-tolerated treatment for the management of Hurley stage II–III Hidradenitis suppurativas [18]. Dalbavancin may have immunomodulatory effects which need more attention.

Compared to linear peptides, cyclic peptides have several advantages. First, it is known that peptide bonds are susceptible to proteolytic degradation, especially under physiological conditions where spontaneous degradation may occur [19]. Therefore, conversion to cyclic peptides improves the in vivo stability compared with its linear form. For peptides with unstable motifs in the sequence, the cyclization process has been shown to prolong the integrity of linear peptides [19]. Second, from a geometric point of view, some cyclic peptides can exert similar biological activity, which mimics the active protein fraction [3]. Third, it was also demonstrated that cyclic peptides enhance receptor subtype specificity. By targeting motifs overexpressed in cancer cells, the binding affinity with their receptor subtypes is enhanced [20]. Specifically, studies have confirmed that cyclic peptides containing cysteine residues have higher binding affinity for target receptors than their linear counterparts [21]. Cyclic peptides were identified as a class of conformationally stable and homogeneous peptide molecules. They possess high metabolic stability, high oral availability and high selective affinity for receptors. Due to the limitation of conformational flexibility of the macrocyclic structure that reduces the entropy value of drug target binding, cyclic peptides’ binding stability was significantly improved, leading to high affinity and recognition specificity between them and the target protein [20]. In addition, due to the compositional characteristics of amino acids, cyclic peptide compounds tend to have low cytotoxicity [22]. Furthermore, it is the ease with which cyclic peptides can be subjected to various modifications, processing and monitoring through which they are easily produced by automated chemical synthesis processes [23]. The metabolism of cyclic peptides is slower because they are highly resistant to proteases and have a longer reservoir effect than their corresponding linear counterparts [24]. Such features allow them to be used to mimic the structure of biologically active peptides, such as peptide hormones, and to bind drug targets in vivo. In conclusion, cyclic peptides and cyclic peptide-like compounds combine several favorable properties. This makes them attractive forms for the development of therapeutic agents. The vast majority of clinically approved cyclic peptides, such as antibacterial agents or human peptide hormones, are derived from natural products. To date, more than 40 cyclic peptide drugs have been approved for clinical use. On average, approximately one novel cyclic peptide drug enters the market each year.

## 3. Natural Cyclic Peptide-Based Antibiotics and Their Functions

The use of cyclic peptides in the development of antibiotics has demonstrated the strength of their stable structure in binding relatively small targets. This can only be achieved with large proteins, as traditionally thought [25]. Thus, as a source of inspiration for designing new antibiotics, natural products based on large cyclic peptides provide medicinal chemists with powerful new antibiotic drugs, drug candidates and scaffolds. The action of cyclic peptides depends on the combination of secondary structure, charge and hydrophobic and amphiphilic properties, unlike the binding mode of small molecules [26]. To date, natural product screening is undoubtedly the most efficient method for the discovery of new antibiotics, such as penicillins, cephalosporins, macromolecular lactones such as erythromycin, glycopeptides such as vancomycin, and teicoplanin, tetracyclines and aminoglycosides.

In the last 2 decades, the Food and Drug Administration (FDA) has approved 6 cyclic peptide antibiotic drugs, some of which are semisynthetic cyclic lipopeptides [27]. The function of these drugs is reflected in their ability to block the transpeptidation of peptidoglycan precursors in the bacterial cell wall by binding to their targets [28]. Because they all contain a common heptapeptide core, five fixed residues serve as the primary binding sites for D–Ala–D–Ala targets. All three antibiotics contain lipophilic side chains. These side chains show the ability to increase the residence time near the target site by anchoring in the cell membrane and/or destabilizing the bacterial membrane [27]. One more function that cannot be ignored is the prolongation of plasma half-life as well, which is due to the interaction of hydrophobic tails with cell membranes and plasma proteins. Therefore, these three drugs are used in the treatment of complex skin and structure infections as well as hospital-acquired pneumonia. Notably, there are subtle differences in the pharmacological effects of cyclic peptide antibiotic drugs due to fine-tuning their activity against different bacterial strains or different pharmacokinetic properties [27].

Daptomycin is a cyclic lipopeptide antibiotic isolated from *Streptomyces roseus* [16]. Its spatial structure is 13 residues in length and carries two nonclassical amino acids, which allow its efficacy to put Daptomycin to be able to act by inserting its decanoic acid into the cell wall of gram-positive bacteria in a phosphatidylglycerol-dependent manner. Subsequently, in the membrane of the target bacteria, the aggregated Daptomycin disrupts the curvature of the membrane and creates pores to induce ion leakage and loss of membrane potential. Ultimately, cell activity is inhibited, and apoptosis is induced.

The cyclic peptide anidulafungin is a member of the antifungal drug class. Anidulafungin, an echinocandin B derivative, is a natural fermentation product of *Aspergillus oryzae* in which the linear acyl side chain tail is replaced by a lipophilic alkoxy triphenyl. Anidulafungin is semi-synthesized from echinocandin B by diacylation to remove the linoleyl side chain and then undergoes three steps, including reacylation with the triphenyl acyl chain [29]. Furthermore, the clinical efficacy of anidulafungin in inhibiting invasive candidiasis occurs through noncompetitive inhibition of β–(1,3)–glucan synthase [30]. In addition, anidulafungin has a low oral bioavailability of 2% to 7% and can be administered nonintestinally [31]. It has a half-life of 27 h and is metabolized to linear peptides, primarily by open-loop hydrolysis.

Caspofungin, micafungin and anidulafungin all belong to the cyclic hexapeptide family. Caspofungin and micafungin were approved in 2001 and 2005, respectively. Their qualities are that they share a similar peptide core consisting of six amino acids, two of which are threonine or threonine derivatives, while the other two are proline derivatives [27]. As a semisynthetic analog of pneumococcal B0, caspofungin is also a naturally occurring lipophilic ring. It was isolated from the fungus *Glarea lozoyensis* and approved in 2001 for the treatment of yeast and fungal infections under specific conditions [32]. It has a half-life of approximately nine days and undergoes mostly ring-opening metabolic hydrolysis and N-acetylation. Micafungin is a semisynthetic analog of the natural cyclic hexapeptide, which undergoes enzymatic deacylation and chemical deacylation of the N-terminal palmitoyl group to give the best N-acyl isoxazole analog [33]. However, unlike the other two members, micafungin has low oral bioavailability and is only for parenteral use with a half-life of approximately 15 h [34]. Most micafungin is metabolized and excreted into the feces via enzyme-modified side chains. Overall, all three antifungal agents are clinically effective against invasive candidiasis and other forms of systemic fungal disease [35].

Currently, the above antimicrobial cyclic peptides are used in clinical treatment cases of pathogenic bacterial infections, wound healing and cancer, but the full-scale promotion of natural antimicrobial cyclic peptides is inevitably plagued by factors, such as source, production cost and biosafety. Compared with direct extraction from organisms, artificial synthesis makes it possible to prepare cyclic peptides on a large scale.

## 4. Man-Made Cyclic Peptide-Based Antibiotics and Their Functions

Similar to approved drugs, most cyclic peptides are natural products of microbial origin or derivatives of human hormones. However, several classes of cyclic peptides in clinical studies have been obtained by de novo design or in vitro evolution strategies [20].

Murepavadin is a *Pseudomonas aeruginosa*-specific cyclic peptide antibiotic. It is used for the treatment of *P. aeruginosa* infections and is an outer membrane protein-targeting antibiotic [36]. After multiple rounds of repetitive peptide library synthesis and screening, one macrocyclic peptidomimetic (Murepavadin) with strong antibacterial activity was discovered, which targets lipopolysaccharide (LPS) transport protein D (LptD) and inhibits LPS transport specifically in *Pseudomonas* spp. [37]. The antimicrobial peptide has a β-hairpin structure with broad-spectrum antimicrobial activity by interfering with membrane cleavage and biosynthesis processes. It has 14 amino acid residues with reduced cell lytic activity but significantly increased antibacterial activity and selectivity against *P. aeruginosa* [36]. In 2014, Murepavadin was approved by the U.S. FDA for the treatment of *P. aeruginosa* infection caused by bacterial pneumonia and was granted expedited review status and qualified infectious disease product status by the FDA [38].

Balixafortide is a bicyclic peptide anticancer compound that acts as a potential C–X–C motif chemokine receptor 4 (CXCR4) antagonist for bone marrow stem cell transplantation and tissue injury [39]. Blocking CXCR4 prevents it from binding to stromal cell-derived factor-1 (SDF-1) ligands, which is essential for stem cell transplantation, tissue regeneration and chemotherapy. Balixafortide was developed through a combination of rational design and a repeat optimization strategy for peptide activity testing. The initial template is polylactide protein II, and the β-folded structure of the compound is first stabilized by the protein epitope mimetics (PEMs) technique with a D–Pro–L–Pro template. The sequence is then optimized in several rounds by combining other properties and using a very different amino acid sequence from the initial template to obtain the final balixafortide. The median half-life of balixafortide is within 10 h. Pegcetacoplan is a polyethylene glycolated cyclic peptide C3 inhibitor that plays an important role in the complement immune system [5]. Pegcetacoplan binds to the C3 convertase binding site on C3, thus preventing its conversion to C3b molecules. In May 2021, pegcetacoplan was approved by the US FDA to treat adults with paroxysmal nocturnal hemoglobinuria (PNH), and it is the first to target C3, a complement component upstream of C5 [40]. The compound was derived from compstatin, a disulfide-bonded cyclized 13-peptide that was screened by a phage display library. ALRN-6924 is a stabilized, cell-permeating antitumor peptide that disrupts p53 inhibition through mouse double minute 2 (MDM2) and MDMX and thereby restores normal P53 function and allows tumor cells to enter the apoptotic process in TP53-wild-type (WT) tumors [41]. Structural optimization of this compound increased the cellular penetration of the drug in addition to increasing the stability of the cyclic peptide. The synthesized peptide mimics the α-helical structural fragment on the p53 molecule used to bind MDM2/MDMX. ALRN-6924 is currently undergoing phase 1/2 clinical studies in patients with advanced solid tumors or lymphomas expressing wild-type p53 and phase 1 clinical studies in patients with AML or advanced myelodysplastic syndromes [24]. In the latest study progress presented at the 2017 American Society of Clinical Oncology (ASCO) meeting, ALRN-6924 had a 59% disease control rate in 41 patients with solid tumors without TP53 mutations, with two patients in complete remission. Crucially, these drugs achieved a median treatment period of 180 days, with encouraging progress in patients who had been taking the drugs for more than 2 years.

## 5. New Approaches for Cyclic Peptide Discovery

The new era of cyclic peptide drug discovery is at the forefront of modern medicine. According to the FDA database, over 60 cyclic peptides have entered the clinic, and approximately one cyclic peptide drug enters the market every year [42]. However, most of the clinically approved cyclic peptide antibiotic drugs are still derivatives of natural cyclic peptides (Table 1). Various sophisticated screening techniques, including phage display, mRNA display, split-intein circular ligation of peptides and proteins (SICLOPPS) and computer screening, have been developed to facilitate cyclic peptide discovery.

**Figure 2:**
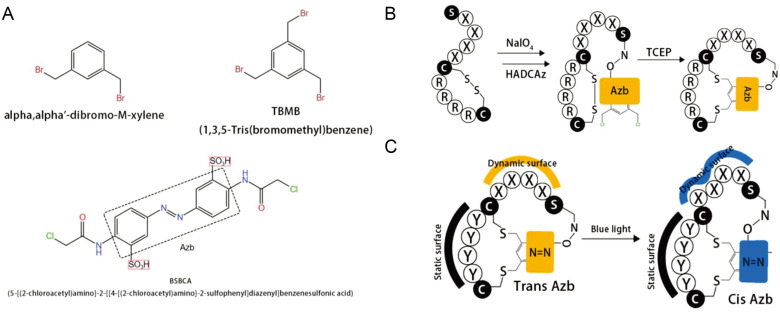
Photoconversion cyclic peptide [44]. (**A**) Structures of the compounds. (**B**) A tridentate C2-symmetric hydroxyl amine and dichlorobenzene containing an azobenzene (HADCAz) LR linker with two orthogonally reactive functionalities. (**C**) Reversible isomerization from the *trans* to *cis* form upon irradiation with blue light (365 nm).

The basic idea of displaying peptides on a phage, introduced by George P. Smith in 1985, was awarded half of the 2018 Nobel Prize in Chemistry for this pioneering work. The earliest preparations of cyclic peptide libraries actually relied on a combination of phage display and chemical methods [43]. The two cores of phage display technology are library building and screening. Different exogenous genes are inserted separately into the phage vector, and the exogenous genes are expressed along with the shell protein so that the peptide or protein is displayed on the phage surface as a fusion protein, which can maintain its relative spatial structure and biological activity [20]. The linear library can then rely on its own cysteine to be linked to the reactant and thus cyclized. Screening refers to the selection of specific antibodies against an antigen from the antibody library and is a key part of the process of obtaining high-affinity antibodies. Commonly, solid-phase screening, liquid-phase screening, cellular screening, tissue section or in vivo screening, selective infection screening and protein microarray screening are available [20]. Some of the most cutting-edge cyclized molecules come from the Ratmir Derda lab. They synthesized light-responsive (LR) bicyclic macrocycles from linear peptides composed of 20 natural amino acids [44]. Their bicyclic peptide contains two amino acid rings in which the azobenzene part can change from trans to cis isomerism in response to 365 nm blue light (Figure 2).

**Figure 3:**
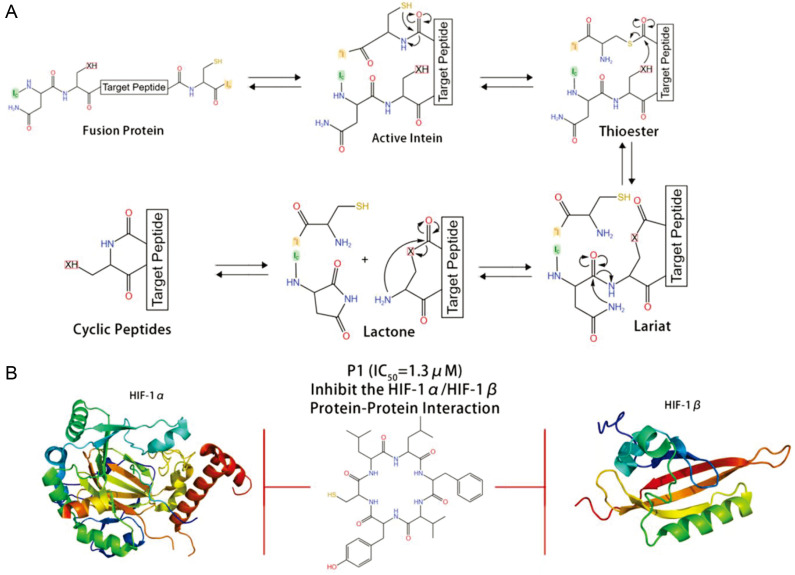
SICLOPPS for macrocyclic peptide synthesis and discovery. (**A**) Schematic diagram of SICLOPPS. (**B**) A cyclic peptide inhibitor cyclo-CLLFVY that targets the HIF-1α/HIF-1β dimer [46].

SICLOPPS is another technology for backbone macrocyclic peptide synthesis and a novel method for sieving functional cyclic peptides in cells [45]. This method inserts the target peptide into an intron. After gene expression, the intron is spliced, cyclizing the target peptide and forming a peptide library (Figure 3). From a library of 3.2 million cyclic hexapeptides, Tavassoli’s lab used SICLOPPS to discover the cyclic peptide inhibitor cyclo-CLLFVY that targets the hypoxia inducible factor-1 dimer (HIF-1) with selectivity for the HIF-1α/HIF-1β dimer [9,46].

The third technology for cyclic peptide discovery is mRNA display. In 1997, Roberts and Szostak discovered that polypeptides could be linked to the corresponding coding RNA by cyclization [47]. The DNA encoding the target peptide generates a transcriptional translation system with ribosomes, tRNAs and transcription factors; then the nascent ribosomally translated peptide is covalently linked to the corresponding coding RNA by puromycin. Afterwards, the peptide-RNA library is chemically cyclized and finally screened for amplification [48].

The random nonstandard peptide integrated discovery (RaPID) platform was proposed by the Hiroaki Suga group [49]. This method utilizes flexible in vitro translation (FIT) to obtain macrocyclic peptides containing nonprotein amino acids [50]. In FIT, a nuclease is manually screened for the ability to bind tRNA to a variety of nonprotein amino acids. This binding allows ribosomal incorporation of multiple specific nonprotein amino acids, including α-hydroxy acids, N-methyl, D-β-amino acids and amino acids with nonstandard side chains. The RaPID platform then allows for rapid and efficient screening of cyclic peptide compounds against specific targets [51].

**Figure 4:**
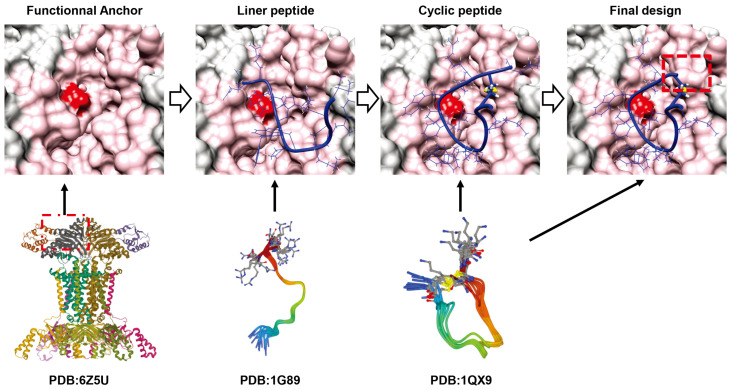
Example of cyclic peptide design. PDB:6Z5U, Cryo-EM structure of the *Acinetobacter baumannii* maintenance of lipid asymmetry BDEF system (MlaBDEF) complex bound to the ATP analog AppNHp [55]; PDB: 1G89, Structure of the bovine antimicrobial peptide indolicidin bound to dodecylphosphocholine and sodium dodecyl sulfate micelles [56]; 1QX9: The three-dimensional structure of cycloCP-11, an indolicidin peptide analog [57]. For the final design step, the K15 residue was removed.

De novo design of cyclic peptides using in silico screening shows great potential [20]. As shown in Figure 4, De Novo Design utilizes the extensive research background and structural biology knowledge of the past decades to optimize various variables of proteins by computer, including sequence composition and structural features as well as folding profiles [52]. Unlike previous iterative and repetitive biological screens, computerized screens utilize empirical signature structures and validated findings to optimize and design key components [20]. Most superbugs with antibiotic resistance carry bla(NDM-1). NDM-1 is capable of hydrolyzing β-lactamase antibiotics. There are several examples of conventional drug design where competitive inhibitors are designed by modifying the structure of natural substrates [52]. Therefore, attention was turned to the binding mode of β-lactamase antibiotics to NDM-1. For example, some macrocyclic peptides have been constructed from a mixture of L- and D-amino acids (D-type structures tend to have stronger affinity) [53]. The design of another group is similar to that of Mulligan in that it uses a portion of the natural product largazole as the "anchor" for cyclic peptide design, targeting the cancer target histone deacetylase [54]. In particular, long alkyl thiols of largazole can penetrate deep into the Histone Deacetylase (HDAC) binding pocket and can be coordinated with the catalytic Zn^2+^ ion, which binds to the unnatural amino acid (2S)-2-amino-7-thioalkylheptanoic acid (SHA). Importantly, the rational design of a potent peptide, des4.3.1 (IC_50_ = 17 nM), was indicated. It is 88-fold more selective for Histone Deacetylase-6 (HDA6) than for Histone Deacetylase-2 (HDAC2), which overcomes the difficulties in selective targeting posed by the high sequence homology of the HDAC family [54].

## 6. Conclusions

It is understood from the above that cyclic peptides are stable peptide analogs with strong conformational stability, ease of synthesis, high specificity, affinity and biostability [36,38]. Thus, both cyclic peptides and cyclic peptide antibiotics are at the forefront of modern drug discovery efforts. As attractive molecular backbones, the versatility of the cyclic peptide backbone gives it a wide variety of functions that can challenge a variety of challenging targets, even traditional small molecules cannot [8]. New and powerful technologies based on rational design and in vitro evolution have enabled the redevelopment of cyclic peptide AMPs for which nature has not provided a solution. Studies of cyclic peptide antibiotics currently undergoing clinical evaluation suggest that new sources of such cyclic peptide ligands are bringing novelty to the field. As chemical synthesis methods are continuously updated and screening techniques become more efficient, it is likely that more structurally novel and more active cyclic peptide antibiotics will enter clinical trials in the near future.

## Figures and Tables

**Table 1:** Cyclic peptide derived antibiotics approved by the FDA in the last 20 years *.

Year	Generic/Trade Name	Target	Indication
2005	Micafungin/Mycamine	1,4-β-glucan synthase	antifungal
2005	Daptomycin/Cubicin	Membrane pore formation	antibacterial
2006	Anidualafungin/Eraxis	1,5-β-glucan synthase	antifungal
2009	Telavancin/Vibative	Cell wall synthesis	antibacterial
2014	Dalbavancin/Dalvance	Cell wall synthesis	antibacterial
2014	Oritavancin/Orbactiv	Cell wall synthesis	antibacterial

* https://www.fda.gov/drugs (accessed on 1 November 2022) for more information.

## Data Availability

Not applicable.
